# Preliminary assessment of the safety factors in K-DEMO for fusion compatible regulatory framework

**DOI:** 10.1038/s41598-022-12389-w

**Published:** 2022-05-18

**Authors:** Beom Seok Kim, Suk-Ho Hong, Keeman Kim

**Affiliations:** 1 Department of Mechanical & Automotive Engineering, Seoul National University of Science & Technology, 232 Gongneung-ro, Nowon-gu, 01811 Seoul, South Korea; 2 Korea Institute of Fusion Energy, 169-148 Gwahak-ro, Yuseong-gu, 34133 Daejeon, South Korea; 3 Korea Institute of Energy Technology, 200 Hyeoksin-ro, Naju, Jeollanam-do 58330 South Korea

**Keywords:** Nuclear fusion and fission, Nuclear waste, Mechanical engineering, Experimental nuclear physics, Magnetically confined plasmas

## Abstract

We open an avenue for discussing how we can pave the way for compliance with existing regulations is a far-reaching factor for settling nuclear fusion technology. Based on a model of the Korean Fusion Demonstration Reactor (K-DEMO) with a target fusion power of 2.2 GW, we assess the intrinsic safety determinants of internal energy sources, the expected radioactive waste, and the tritium management. Regarding these safety factors, we scrutinize the compatibility of the current legislative environment in Korea with K-DEMO and envisage foreseeable obstacles, such as licensing of the nuclear facilities and acceptability of the radioactive waste. Based on precedent licenses for the Korean Superconducting Tokamak Advanced Research (KSTAR) and lessons learned from the International Thermonuclear Experimental Reactor (ITER), we examine hazardous factors that would threaten regulatory compliance of K-DEMO. This approach can help shape a fusion-compatible framework for consolidating the necessary technical provisions and regulatory baselines reflecting social acceptance with a sense of safety. Fusion-compatible aspects in the regulatory environment are discussed, from fusion philosophy to subordinate administrative and technical guidelines, facility classification, and detailed methods guaranteeing integrity and safety. This paper will contribute to the timely settlement of fusion demonstration facilities and subsequent commercial plants.

## Introduction

Cutting carbon and greenhouse gas emissions is currently at the forefront of global energy and environmental issues. Under this background, nuclear fusion technology that does not emit carbon at all and does not require any fossil fuels is attracting attention as a next-generation energy solution^1–3^. In parallel with efforts to achieve 2050 carbon neutrality, nuclear fusion research and development for future energy is accelerating as a long-term mission^4–7^. In addition, private start-ups have appeared and are keeping pace with this^3,8^. If the institutional acceptance of society is secured, with such technological advances, the implementation of fusion power generation will be promoted^9^.

The legal system, which is laid on the consensus of society, is an indicator of the public’s acceptance of novel technology in terms of technical acceptability and socio-environmental compatibility. In Korea, where the Korean Superconducting Tokamak Advanced Research (KSTAR) project^10,11^ was completed, and is still in operation today, the first legal base of the fusion energy development was set via the statute of “Fusion energy development promotion act” enforced in 2015^12^. Additionally, the statute entitled “Act on the establishment, operation and fostering of government-funded science and technology research institutes, etc.” came into force in 2020^13^. These legislations clearly specify the legal status of the Korea Institute of Fusion Energy as a major player in nuclear fusion research. Moreover, beyond the progress of the International Thermonuclear Experimental Reactor (ITER)^5,14^, the Korean government solidified its willingness to foster nuclear fusion development to advance the dream of a nuclear fusion plant.

On the other hand, the institutional system includes standards and regulations. In particular for demonstration facilities^6,7,15–17^ that will be realized prior to the implementation of commercial fusion power plants, establishing a fusion-compatible regulatory environment is necessary. It can be seen that when clear classification criteria for fusion facilities and preparation of licensing evaluation factors are not prepared, considerable social costs might be consumed. For example, it took a total of 7 years for KSTAR’s licensing process^18,19^, and this can be attributed to the lack of appropriate regulations for fusion facilities at that time. Because no country has a specific fusion-compatible regulatory framework to settle issues from siting to decommissioning^9^, preparing regulatory measures is required based on a clear identification of objectives and assessment of associated safety factors by closing the gap between new technological requirements and the existing framework^9,20^.

The aim of this paper is to extend the discussion on whether the current nuclear regulatory landscape can meet the requirements for an upcoming fusion demonstration facility of the Korean Fusion Demonstration Reactor (K-DEMO) and level the playing field for the future demonstrations. In this study, we predict licensing barriers for K-DEMO under the current Korean nuclear regulatory framework, which has no fusion-specific compatibility. This preemptive analysis will reveal the philosophical differences between fission and fusion and the technical- and administrative constraints for K-DEMO. Based on the primary design factors selected in a conceptual model of K-DEMO, key design elements related to the purpose and scale and the consequent technical requirements are examined at the project level. Moreover, energy source terms were quantitatively analyzed. Using the results, we scrutinize the internal energy sources based on the recognition that this facility is not a simple installation but more comparable to a quasi-commercial power plant. In addition to licensing issues, we evaluate the radiation characteristics of expected waste from K-DEMO in accordance with the existing fission-based regulatory framework.

## Conceptual model of K-DEMO and analysis methods

In this study, we considered the conceptual K-DEMO model to generate 2.2 GW of fusion power in the initial stage. The tokamak has a major radius of 6.8 m and a minor radius of 4.2 m^15,16,21–23^. It equips with vertically symmetric double null divertors and breeding blankets for self-sustainable tritium breeding and neutron multiplication during the operation. A Rankine cycle using water as the main working fluid was applied in the energy conversion process from fusion-, thermal-, and electricity. To evaluate the compatibility with the domestic regulatory framework, the licensing barriers of K-DEMO were analyzed first. In addition, we performed the characterization of expected radioactive waste to evaluate its acceptability under the current framework. The current Korean Nuclear Safety as of 2021, which provides a prescriptive and conservative approach, was referred to. The difference with K-DEMO was revealed by analyzing the imposition and procedure of the current laws and regulations at the time of the licensing process of KSTAR and ITER.

Accounting for the radioactivation characterization, we analyzed the activation level and decay heat generation from the divertors and blankets, which were conservatively assumed to be exposed to the fusion neutrons during two years’ continuous full-power operation^16,18,21^. The calculation procedure was divided into two stages: identifying the operating conditions and design configurations and performing a radioactivity analysis. For the first stage, we calculated the source terms, identified the significant constituent materials of each component, and established specification and cooling scenarios for the facilities. In the second stage, we used the FISPACT code to calculate radioactivation and solid waste emissions^21,24–27^. We performed a Monte Carlo neutron particle transport code (MCNP) simulation to derive the neutron spectrum and flux to calculate the source terms. For the local neutron wall load (NWL) profile as an activating source corresponding to the full power of 2.2 GW, we assessed it using a developed in-house code^21^, and projected the profile to plasma-facing surfaces of in-vessel components of blankets and divertors. Accounting for this profile, we analyzed the radioactivation level and decay heat generation of the water-cooled ceramic breeder blanket and divertor by the in-house code utilizing a nuclide emission data library. Through the neutron transport analysis using MCNP, the neutron flux is derived for each layer in the 1-D model according to refined energy levels from 10–10 to 14.1 MeV^24,27^. The blanket and the divertor components are handled as 1-D layered models. The detailed features of the 1-D models for the analysis are schematically presented in Supplementary Information^21,28^. During the evaluation of the simplified 1-D models, homogenized features and properties were projected in volumetric aspects regarding the reliable activation assessment. A 1-D blanket model consists of a W layer, an adhesion layer of vanadium, structural material of reduced activation ferritic martensitic (RAFM) steel and the pebble beds of Li_4_SiO_4_ and Be_12_Ti, which are tritium breeder and neutron multiplier, respectively. A 1-D divertor comprises is composed of W mono-blocks, RAFM heat sinks, and a cassette body. The radioactivation characteristics were assessed using MCNP by separately interpolating the effects of energy dissipation (decay) of neutrons in the direction of neutron penetration (i.e., the radial direction in the tokamak) in the volumetric structures. The radioactivity concentrations and decay heat characteristics were evaluated as a function of the time and position of each constituent element of the structure.

## Regulatory scope: licensing of fusion systems

### System configurations and corresponding licensing procedures: KSTAR and ITER

**Figure 1:**
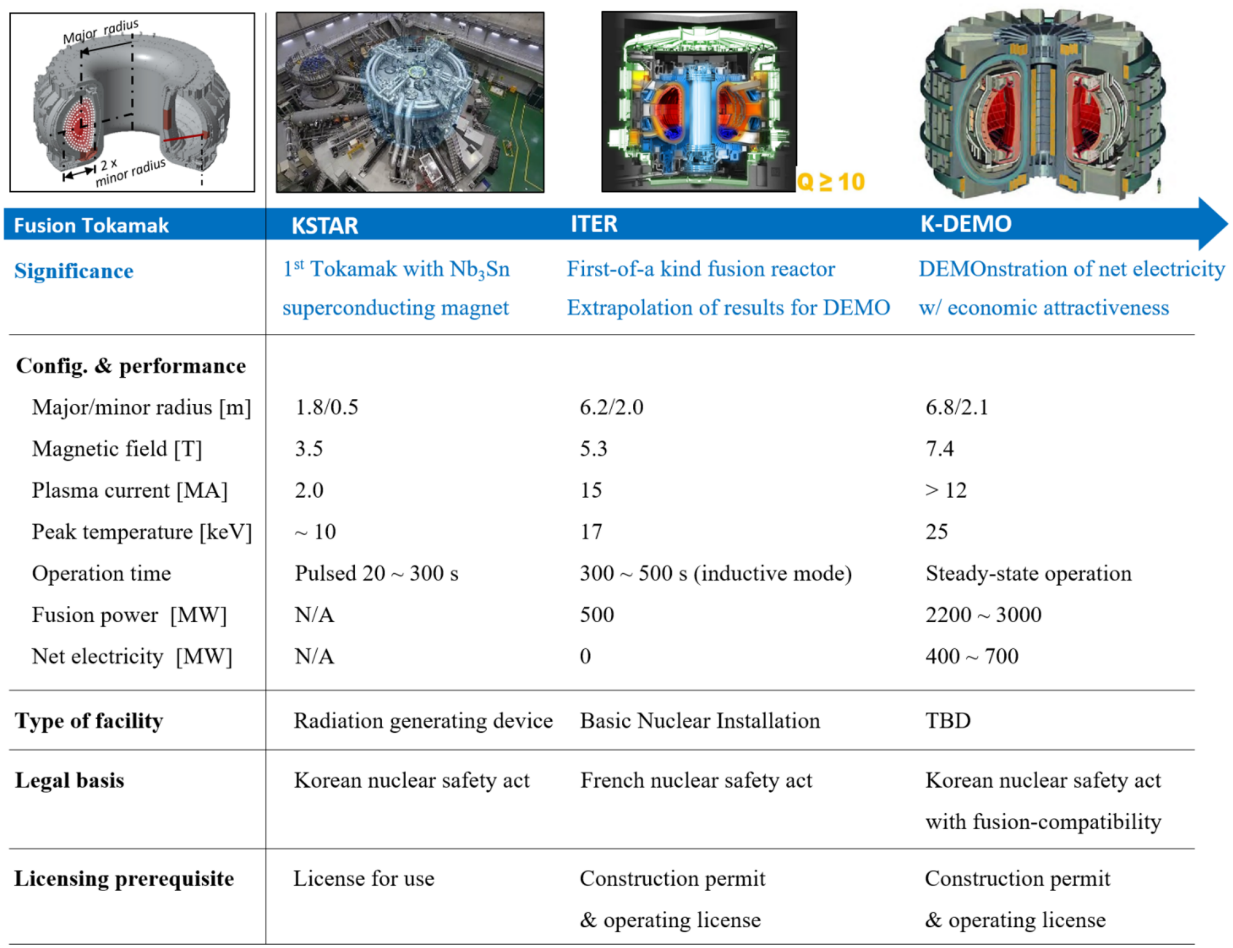
Key details of the representative fusion facilities and licensing requirements under local regulatory frameworks.

Korea acquired technological experience and knowledge on nuclear fusion installation through the successful construction and operation of KSTAR. In addition, Korea is a significant stakeholder in ITER project. Currently, conceptual research on K-DEMO is underway. Figure 1 shows representative specifications for K-DEMO along with a comparison of the purposes and specifications of KSTAR and ITER^10,16,19,29–32^. K-DEMO is expected to generate approximately 2.2 GW of thermal power and supply 4–700 MW of net electricity when the Rankine cycle is applied at current levels of technology. Superficially, it is slightly larger in size (major radius of 6.7 m) than ITER (6.2 m) and has a plasma current of 12 MA or more. ITER, K-DEMO and KSTAR differ significantly in terms of their purpose, scale, system complexity, fuel, load conditions, and consequent safety measures. ITER is intended to establish safe technological extrapolations, and K-DEMO is intended to demonstrate an economically attractive net electricity gain. In addition, K-DEMO is expected to operate on a significantly larger scale and with a greater system complexity than KSTAR. Therefore, licensing requirements should be reviewed for their applicability.

KSTAR was the first nuclear fusion research facility that obtained a license under the legal framework in Korea. KSTAR is a facility for nuclear fusion experiments that uses deuterium but not tritium. Regarding its license, the regulatory authority looked at the neutron generation rate by deuterium fusion as a principal factor, which should not to exceed 1.5 × 10^17^ per shot and 1.2 × 10^20^ per year as presented in Table 1. The conceptual research and design of KSTAR, which comprises a vacuum vessel, cryostat, magnet structures, current drive facilities, and auxiliary installations, were initiated in 1995, and the facility was completed in 2007. It entered the regulatory purview of the Nuclear Safety Act during its construction phase. As a radiation-generating device, administrative and technical reviews began in 2000 according to licensing requirements. After 7 years of review, a permit was finally issued for KSTAR to operate. A compromise was then made under the regulatory scope at the time to permit the use of KSTAR because there were no specific categories or licensing procedures for a fusion facility. The regulations were complemented by the addition of a subsection to the compulsory surveillance tests for operational safety issues. However, there have not yet been any significant revisions or additions to the statutory criteria for licensing conceivable fusion facilities, including demonstration reactors (DEMOs).

**Table 1:** KSTAR specifications and licensing criteria^18,19^.

Specifications	Fusion experiments using deuterium
	No tritium use
	Fusion plasma current < 2 MA
Classification	Radiation generating device by accelerating charged particles (e.g., accelerators)
Licensing criteria	Neutron generation rates less of than 1.5 × 10^17^ per shot and 1.2 × 10^20^ per year

ITER has become the preemptive example of a fusion facility owing to the many years of preparation of its license. No country including France, where ITER is located, has a dedicated regulatory framework for fusion facilities as of 2021^9,20^. Nevertheless, as a *goal-oriented* approach for an international milestone of ITER, it has been authorized for construction as a Basic Nuclear Installation (INB) in France since 2012. ITER is now going through a ‘graded approach’ for the establishment of a regulatory step. Progress is being made in stages, from licensing the first plasma (with hydrogen-helium plasma for the non-nuclear phase) to commissioning in advance of the first use of tritium for the deuterium-tritium (D-T) operation phase^14,33^.

### Foreseeable licensing barriers for K-DEMO under a prescriptive framework

Licensing is a critical issue that will enable fusion technology to gain acceptance by society. Currently, international safety and regulatory guidelines for nuclear fusion are being drafted. We conducted a preliminary study on the regulatory barriers that K-DEMO will face within the current regulatory framework in Korea. First, we examined how K-DEMO would be classified as a nuclear facility based on its major energy sources. Table 2 presents the results of the nominal energy sources and decay heat of K-DEMO, which are compared with those of ITER and the commercial fission plant APR1400^16,18,28^. Because ITER was not originally intended to demonstrate net power generation, K-DEMO has larger energy source values with the core energy of 2–3 GW and net electricity of 400–700 MW. In contrast, ITER has core energy of only 500 MW. K-DEMO performs comparably to the APR1400 with the same order for the nominal power sources. This superficial energy comparison indicates that K-DEMO may be treated as a quasi-commercial power plant.

**Table 2:** Nominal power and decay heat evaluation for K-DEMO and a comparison with ITER and a commercial nuclear power plant (APR1400 model).

Energy sources	Fusion		Fission plant (APR1400)
	K-DEMO	ITER	
**Nominal power**	Fusion to thermal		Fission to thermal
Core energy (MW)	2200–3000	500	4000
Net electricity (MW_e_)	400–700	N/A	1200
**Decay heat**	From in-vessel components		From fuel
Just after shutdown (MW)	63.3	11	316
1 day after shutdown (MW)	7.0	0.6	
1 month after shutdown (MW)	2.5	0.3	–

**Figure 2:**
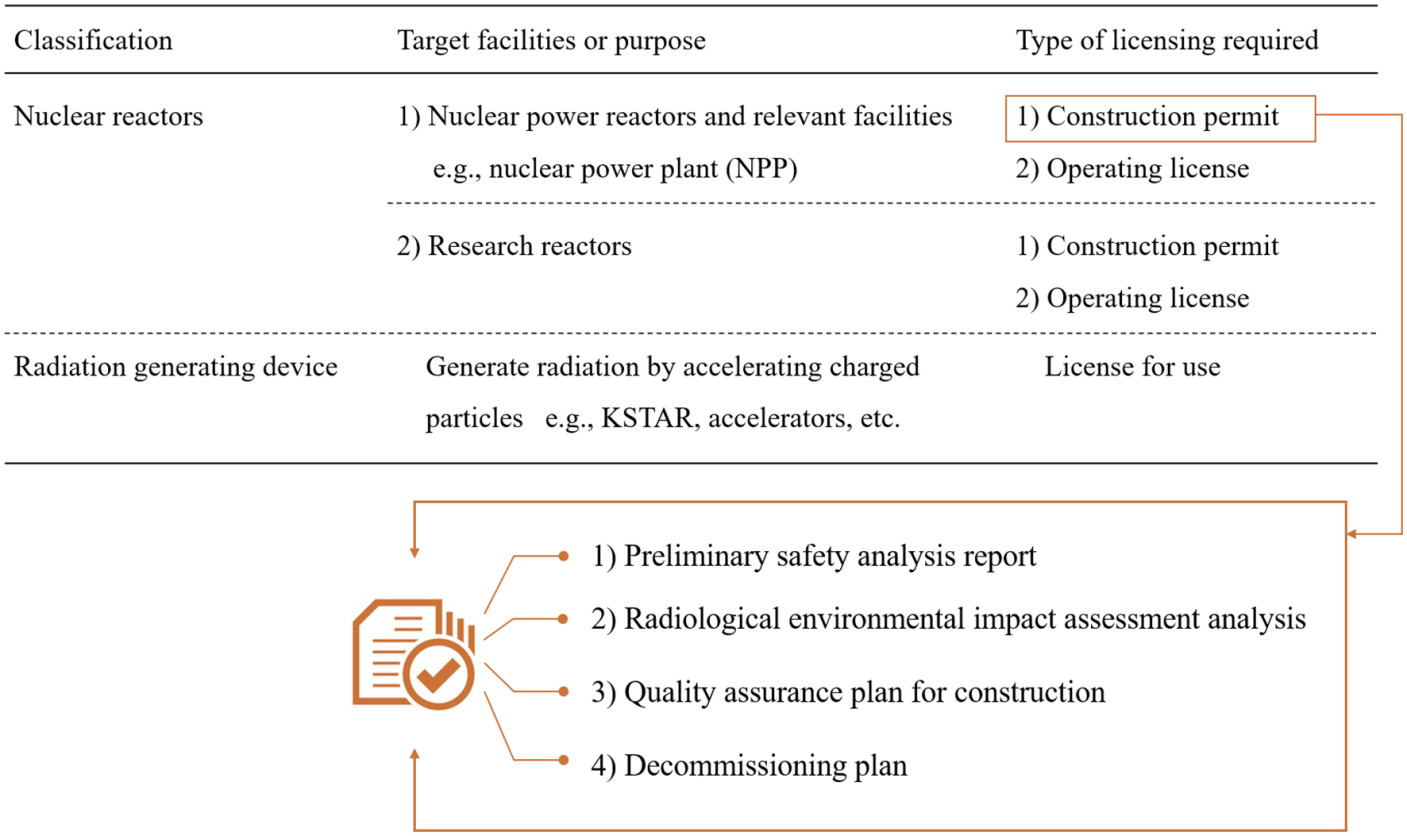
Categories of nuclear facilities and corresponding license requirements prescribed by the Nuclear Safety Act of Korea.

Figure 2 presents the categories of nuclear facilities and corresponding license requirements prescribed by the current Nuclear Safety Act in Korea. Depending on the design, capacity, and purpose, a nuclear facility may be classified as *power-generation facilities* (i.e., nuclear materials are used as fuel) or *radiation-generating devices*, (i.e., accelerates charged particles). The former category is further divided into nuclear reactors and relevant facilities or research reactors. The administrative and technical baseline licensing requirements are determined according to these categories for identifying safety issues and criteria to manage a facility throughout its lifecycle. The construction permit and operating license for power-generating facilities such as nuclear power plants are separate but sequentially applicable to secure a stepwise progressive approach. Based on the core energy (see Table 2), K-DEMO may be asked to comply with the current legal requirements for nuclear power plants. A power-generating facility must meet administrative and technical requirements to obtain a construction permit. In particular, Korea’s Nuclear Safety Act and sub-enforcement ordinances apply a prescriptive regulatory approach, where even the details that need to be described by the required documents are specified. Figure 2 presents the essential requirements. Among them, the preliminary safety analysis report should identify the purpose and detailed specifications of components involving core design, fuel system design, reactor materials, reactivity control system, cooling system, reactor vessel, and ancillary equipment. The current legal framework of Korea is comparable with fission systems, and it was established based on the international guidelines enacted by the International Atomic Energy Agency and International Commission on Radiological Protection, as well as the regulatory frameworks of other countries^34–39^. Preemptively preparing a fusion-compatible regulatory framework is indispensable for the development of a fusion demonstration facility. As a starting point for regulatory preparation, Korea is an active participant in the IAEA-led discussion on identifying fusion safety requirements, transferability of fission-compatible regulations to fusion, and novel guidelines for fusion. The preparation of international guidelines can be used as leverage to improve the local regulatory environment.

## Prerequisites for fusion regulation: identification of safety and hazardous factors

A prerequisite to preparing fusion-compatible guidelines is defining a fusion facility and the associated safety and hazardous factors. Based on the conceptual configuration of DEMOs, researchers have assessed the safety factors of the system and investigated the transferability of the current fission-based regulatory framework to fusion^6,9,32,40^. Developing fusion-compatible regulation for the unprecedented K-DEMO is a prerequisite for securing its social acceptance and sense of safety. Here, we evaluate the energy source, expected radioactive waste, and tritium usage of K-DEMO in detail, which are vital for determining the safety requirements and risk factors.

### Energy source terms in K-DEMO

The principal safety factors of K-DEMO were assessed according to the proposed configuration in the conceptual phase. Table 3 presents our tentative assessment of the energy sources in K-DEMO and a comparison with those of the Japanese DEMO (JA-DEMO) and ITER^7,28,30,41,42^. JA-DEMO and K-DEMO involve significantly larger amounts of energy than ITER. The fusion plasma and magnet structures of K-DEMO are estimated to store 1.08 and 144 GJ, respectively^43,44^. Furthermore, the JA-DEMO and K-DEMO magnets are estimated to store 140% and 188% more energy, respectively, than ITER. This is because stronger magnetic confinement is a prerequisite for higher fusion power to meet the Lawson criteria for plasma ignition. JA-DEMO and K-DEMO are expected to have fusion power levels of 1.35 and 2.0 GW, respectively^30,42^.

**Table 3:** Tentative assessment results of energy sources in K-DEMO and a comparison to those in ITER and JA-DEMO.

Energy sources	ITER	JA-DEMO	K-DEMO
**Plasma**			
Thermal energy (MJ)	350	870	620
Magnetic energy (MJ)	400	450	461
**Magnet**			
Magnetic energy (GJ)	50	120	144
**Chemical potential energy**			
W-steam reaction (GJ)	–	200	138
**Decay heat**			(BLK/DV)
Just after shutdown (MW)	11	38	63.3
1 day after shutdown (MW)	0.6	8.3	7.0
1 month after shutdown (MW)	0.3	2.2	2.5

If the in-vessel components of K-DEMO are assumed to use water as the coolant, then the chemical reaction potential can be conservatively estimated as 138 GJ. The chemical reaction potential should be evaluated to consider the risk of an in-vessel loss-of-coolant accident (LOCA), which involves a complete exothermic reaction between the leaked coolant and plasma-facing material (i.e., tungsten). The chemical reaction potential can be reduced if the design is adjusted to prevent the overall LOCA and mitigate the rapid pressurization of leaking coolant. Applying a compound could help avoid fast chemical reactions with a high-temperature coolant. The primary cooling system should be designed for efficient heat transfer and cooling in the heat exchangers and other relevant components across the entire system. The energy transferred by the enthalpy of the coolant should be assessed through the specific design of an optimized thermodynamic cycle with the aim to supply a net power of 400–700 MW^15,16,45^.

### Relevance of radioactivation of in-vessel components

The radioactivation of structures, particularly in-vessel components such as blankets and divertors, is inevitable because of the high-energy neutrons generated from D-T fusion plasma. With the evolution of fusion systems from experimental models such as KSTAR to large-scale facilities such as ITER and DEMOs, increasing the volume of the tokamak is a physically proven way to secure the required confinement time of the plasma surrounded by magnetic fields^46^. Concerning the size of the fusion tokamak, the geometrical major radius of the torus is in a complementary relationship with the magnetic field of *B*. If increasing the value of *B* is limited by present-day superconducting magnet technology, the ignition criterion for fusion plasma is attained only by expanding the geometric size, which would involve increasing the major radius of the torus^46^. However, this increases the amount of radioactive waste generated, which must be replaced periodically or permanently disposed of. Because there are local regulations for waste regarding its classification and corresponding treatment and disposal methods, the concentration of radionuclides and the decay heat from the in-vessel component should be prioritized as considerations during the design phase.

**Figure 3:**
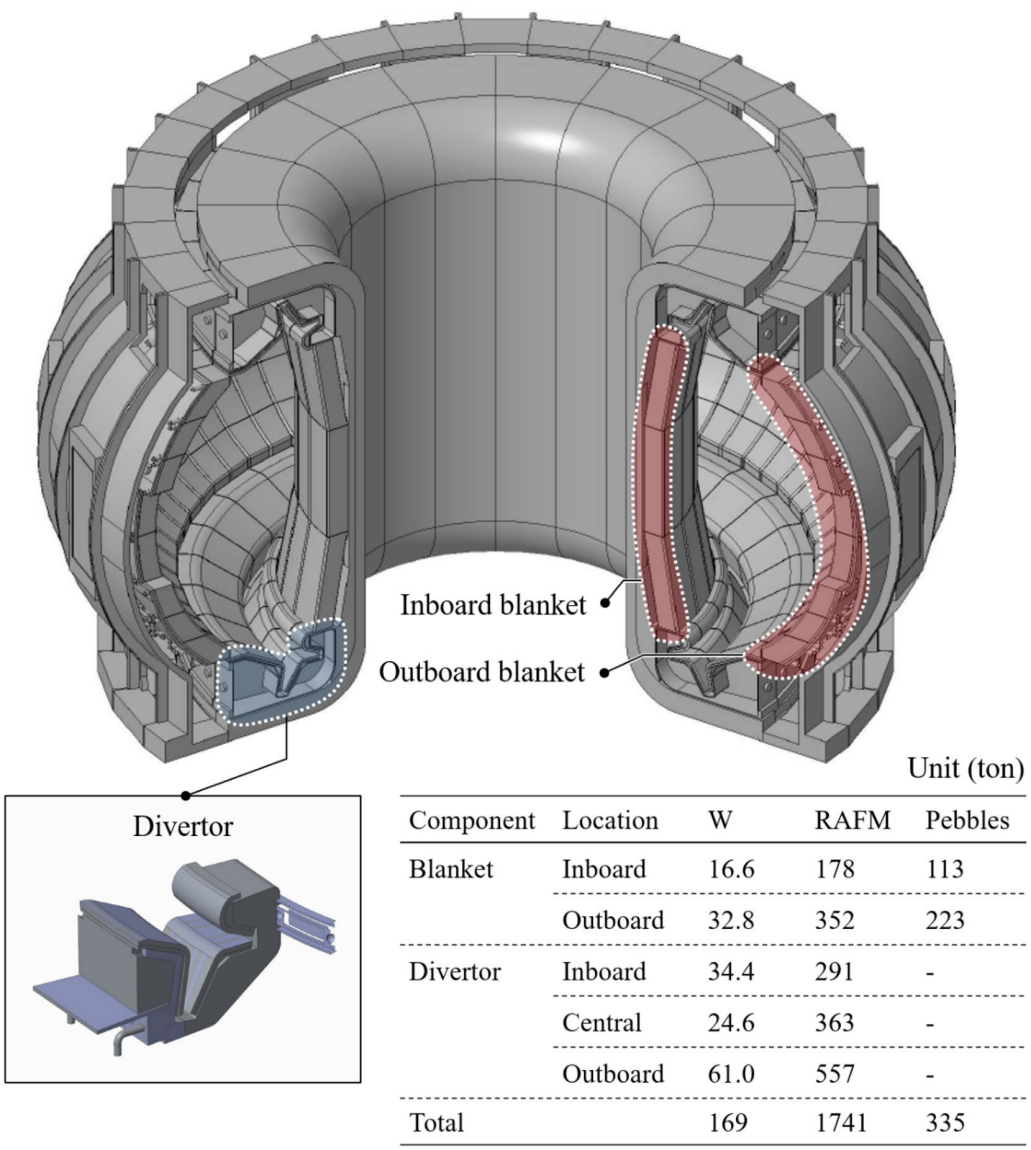
270° cut-view of a conceptual drawing of K-DEMO with a detailed divertor component. The inset table presents the estimated weight of the in-vessel components during a periodic replacement. The functional materials of the breeder (Li_4_SiO_4_) and neutron multiplier (Be_12_Ti) are in the form of pebbles. The structural material is reduced activation ferritic martensitic (RAFM) steel.

**Figure 4:**
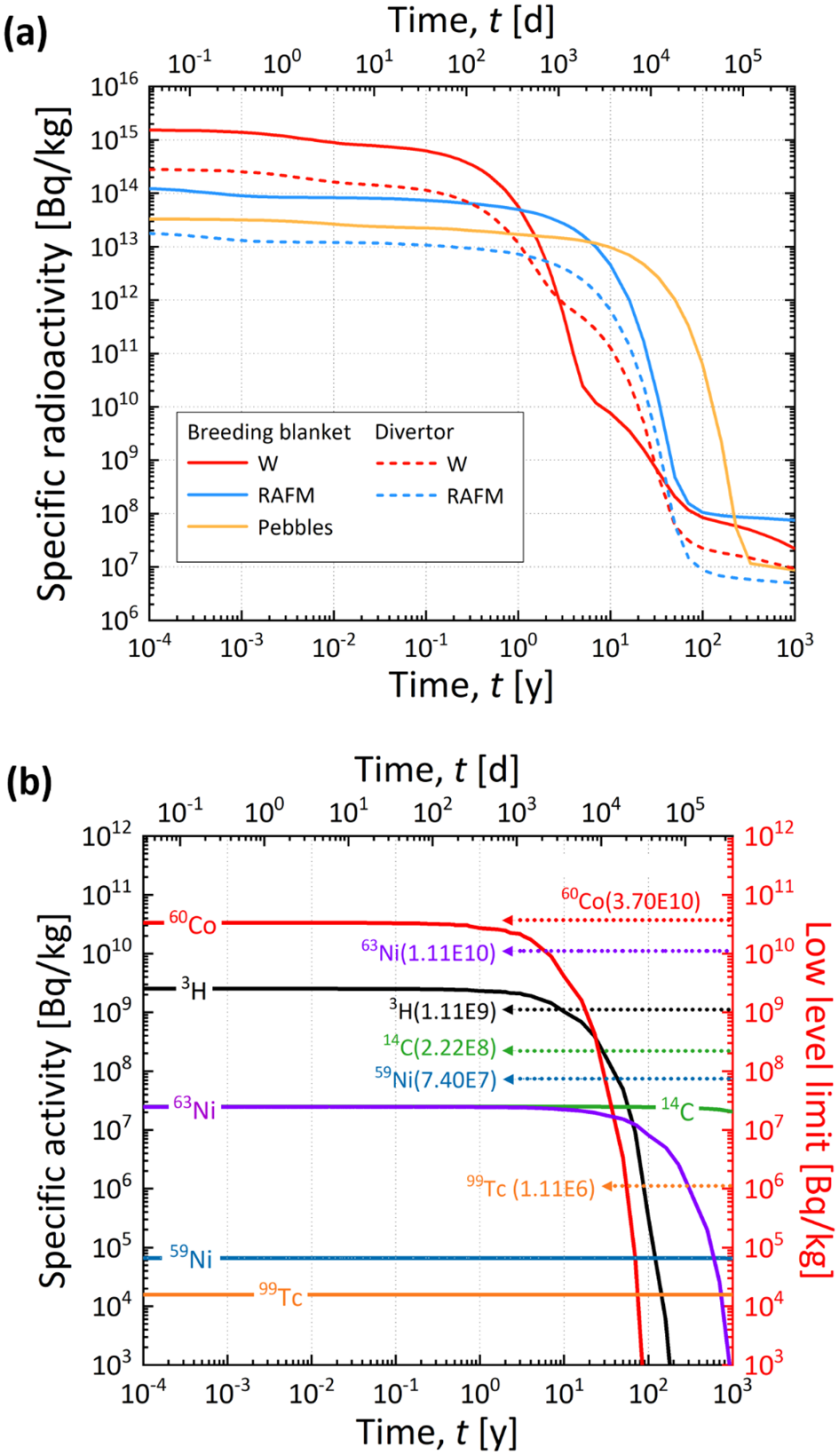
Transient radioactivity of the in-vessel components of K-DEMO: (**a**) Specific radioactivity levels of the breeding blankets and divertor. (**b**) Radioactivity of nuclides from the structural material of reduced activation ferritic martensitic (RAFM) steel and the functional pebble materials of the breeder (Li_4_SiO_4_) and neutron multiplier (Be_12_Ti). Dotted lines from the right of the y-axis indicate low-level limits of the nuclides prescribed by Korean regulations.

**Figure 5:**
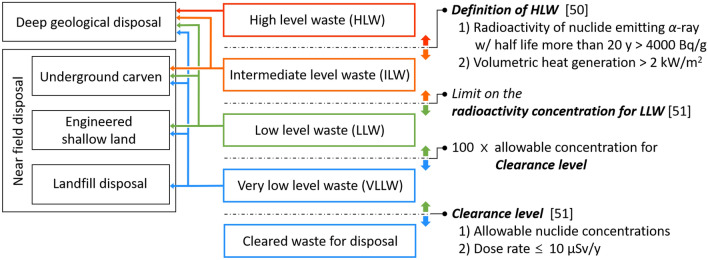
Disposal methods for radioactive waste according to their classification under Korean regulations.

The radioactive characteristics determine the methods required for the maintenance of components and classification of waste^47,48^. The total amount of waste also determines whether there is sufficient hot cell and landfill capacity for its treatment. The disposal of waste should be prepared in advance. Thus, all phases for the construction and operation of K-DEMO must fall entirely within the regulatory framework. In this paper, we point out that determining whether the expected waste can be accepted social is imperative, specifically under the current regulatory framework relevant to radioactive waste. Therefore, we investigated the total amount of waste and its principal characteristics by calculating the sequential radioactivation of major components in K-DEMO under normal operating conditions. Figure 3 shows the assessed solid waste along with the 3-D K-DEMO model used for the analysis, and Fig. 4 shows the corresponding results for the variations in the transient activity of the blanket and divertor. For a conceptual K-DEMO model with a major radius of 6.8, the waste upon replacement of the blanket and divertor was predicted to reach up to 2245 tons, as presented in the inset table of Fig. 3. Figure 4a shows that the plasma-facing components in the blanket and divertor have higher specific radioactivity than other materials just after a shutdown. However, these decrease by 6–7 orders of magnitude within 100 years^21,47–49^. Similar substantial decreases are predicted for most of the materials. The transient radioactivity characteristics presented in Fig. 4b demonstrate that most waste would be categorized as low-level waste with no high-level waste. The current Korean Nuclear Safety Act specifies the waste classification criteria and disposal procedures. Based on social, economic, and environmental aspects, the waste of K-DEMO can be disposed of by shallow burial on land, as shown in Fig. 5^21,50,51^.

**Figure 6:**
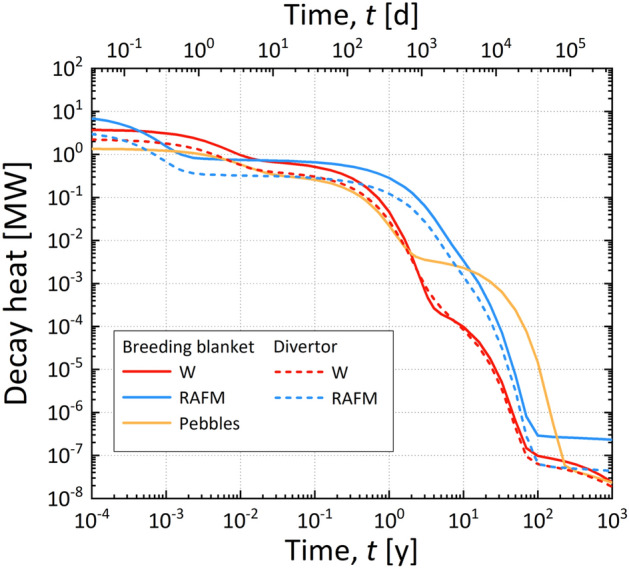
Transient decay heat from the in-vessel components of breeding blankets and divertors in K-DEMO after plasma shutdown. The pebbles (yellow line) are the functional material composing the breeder and neutron multiplier.

We believe that quantifying the risk associated with the radioactivation of structures in terms of safety management and demonstrating that the expected waste is completely disposable within a century will significantly ease the burden of nuclear fusion research. The amount of decay heat and its transient variation, as presented in Table 3 and Fig. 6, respectively, must be carefully controlled to guarantee the engineering soundness of facilities. Suppose the decay heat is not sufficiently taken into account, insufficient cooling may cause a problem in the mechanical safety of the components or related systems due to increased temperature. In particular, for critical replacement components (blankets, divertors, etc.), the transient variation of decay heat after plasma shutdown determines the required cooling method required, the setting of the cooling period for each component, and the sequential replacement schedule accordingly. We expect that significant amounts of waste will be cleared from the category of radioactive waste when radionuclide concentrations decrease below clearance levels and cooling is no longer needed, which introduces the possibility of recycling and reusing materials^47,48^.

### Tritium management

DEMO facilities are expected to consume significant amounts of tritium as fuel. According to the safety assessment of a European DEMO, 125 kg of tritium per year will be consumed to realize a net electric power of 1 GW_e_^40^. We estimated that K-DEMO would have an annual tritium consumption of 123 kg. We should not overlook that KSTAR does not use tritium at all when applying its regulatory scope to DEMOs, which will use tritium. We must secure relevant safety technologies along with an accurate tritium inventory analysis and management strategy. Thus, licensing requirements for tritium management should be clarified according to specifications and the appropriate level of technical reliability^52–55^.

## Regulatory transferability of nuclear safety and relevant engineering soundness

Nuclear safety guidelines for the classification and storage/disposal of waste and the general safety objectives for occupational and public exposure have already been agreed upon by international communities^18,28,35^. Now though the regulatory framework for a fusion facility has not yet been clearly specified in terms of the administrative and technical criteria. A rational approach would be to adopt the philosophy of present regulations to instill a sense of safety as long as no unprecedented radioactive hazards arise due to nuclear fusion. Note that the risks faced by individual workers at fusion plants should be controlled based on safety criteria equivalent to those for different types of plants, and the facilities should not disturb the ordinary lives of the public. On the other hand, reviewing what is recognized as a new risk factor in a large fusion facility will be necessary. For example, the magnetic fields of KSTAR, ITER, and K-DEMO can reach several tesla, as presented in Fig. 1. Therefore, unlike fission power plants, guidelines for ensuring the occupational safety of fusion power plants must address the highly electromagnetic environments.

## Harmonization of codes and standards in a regulatory framework

One way to ensure the engineering soundness of components and systems is to conform to engineering codes and standards (C&S) of relevant fields. ITER uses systematic C&S to guarantee the integrity of its system. Table 4 shows the C&S applied mutatis mutandis to major ITER components’ design and manufacturing process^56,57^. Engineering C&S are appropriately allocated to implement functions and meet safety requirements during the design, fabrication, installation, and construction stages. Herein, C&S were sourced from nuclear engineering, mechanical engineering, materials science, and other fields and applied to specific mechanical components including vacuum vessels, thermal shields, and tritium plants. However, as presented in Table 4, components that were not covered by existing C&S were designed according to the structural design criteria for in-vessel components and technical specifications prescribed by the ITER organization. These included magnet structures, blanket systems, divertors, and test blanket modules^56–58^. Research into new materials is also being carried out to enhance the lifetimes of components to realize yields higher than tens of displacements per atom when used appropriately under fusion conditions. In parallel, attempts have been made to register novel codes for fusion energy devices based on previously proven engineering criteria^31,56,57^. This requires first deriving suitable specifications for fusion systems and defining a set of requirements for preparing and complying with engineering C&S. Unlike simple experimental installations, the design, manufacture, and construction of K-DEMO, which will be exposed to much more severe load conditions than KSTAR and ITER, should be managed through the development and allocation of validated technical C&S baselines over the entire project period.

**Table 4:** Engineering codes and standards for the design and manufacture of major tokamak components of ITER^56,57^.

Components	Assigned codes and standards	
	Design	Manufacturing
Cooling water system	ASME, API, AISC, technical specifications	ASME, API, AISC, technical specifications
Cryostat	ASME, RCC-MR, EN standards, technical specifications	ASME, RCC-MR, EN standards, technical specifications
Divertor	SDC-IC	EN standards, technical specifications
Magnets	MSDC, EN standards	ASME, EN standards, technical specifications
Test blanket system	RCC-MR etc., TBD	RCC-MR etc., TBD
Tritium plant and detritiation	ASME, technical specifications	ASME, technical specifications
Vacuum vessel	RCC-MR	RCC-MR

In addition to codes and standards that have already been developed in relevant engineering fields (e.g., ASMES, API, AISC, RCC-MR, EN standards), specifications established for the design and development stage (e.g., MSDC, SDC-IC) were applies mutatis mutandis as guidelines.

ASME: American Society of Mechanical Engineers; API: American Petroleum Institute; AISC: American Institute of Steel Construction; RCC-MR: Regles de Conception et de Construction des Materiels Mecaniques des ilots nucleaires RNR; EN: European Norm; MSDC: Material Structural Design Criteria; SDC-IC: Structural Design Criteria for In-Vessel Components.

**Figure 7:**
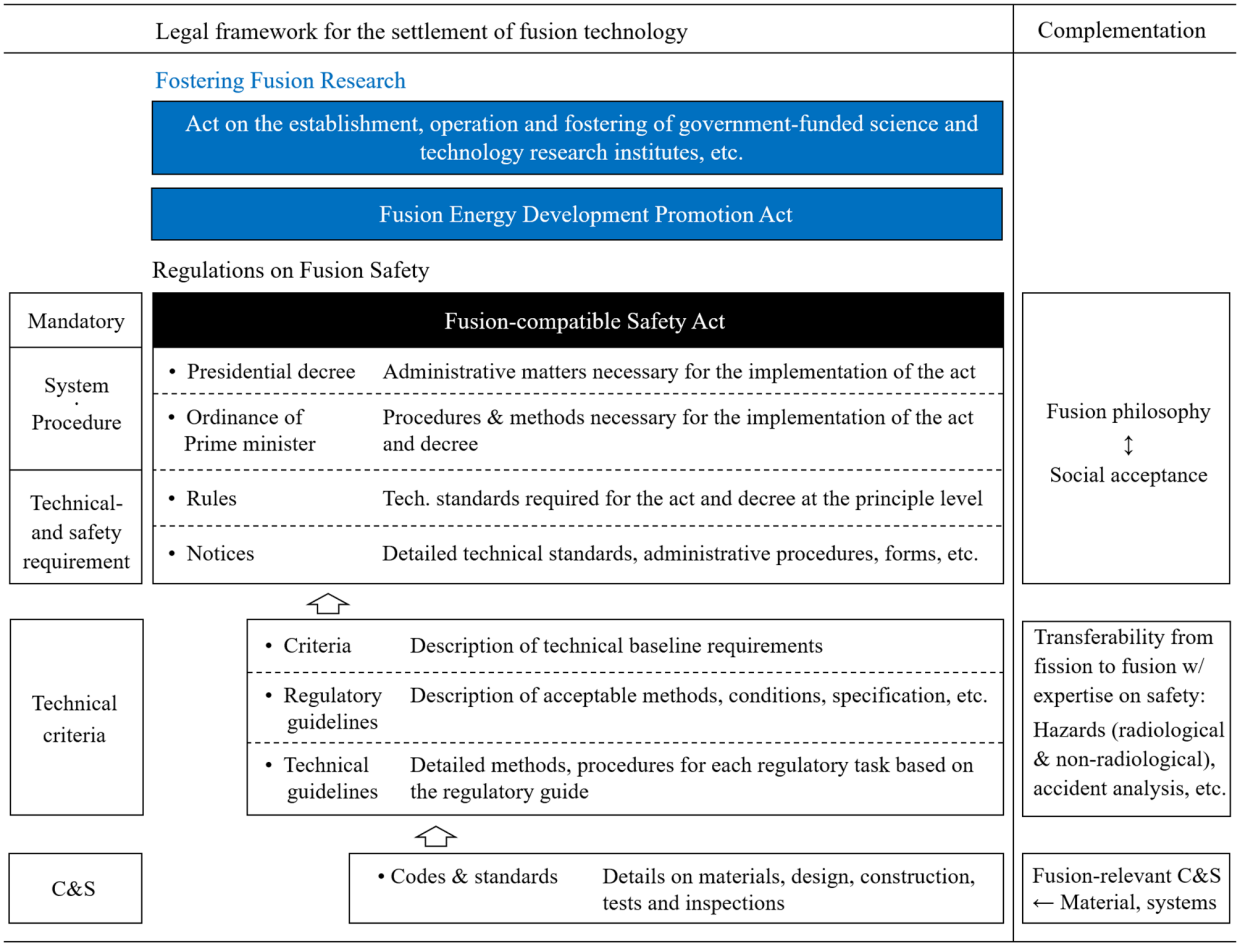
Outline of the proposed legislative environment in Korea to promote fusion research toward the DEMO stage and to impose legal measures to regulate its design, installation, construction, operation, and decommissioning.

It is also essential to harmonize existing safety standards with the intrinsic characteristics of novel fusion facilities. It should be possible to secure engineering soundness without violating nuclear- and other safety standards. Figure 7 outlines a possible regulatory framework that would enable legal and institutional support for developing fusion technology in Korea. As depicted on the right side, the preparation of such a framework should first be accompanied by a clear identification of the purpose, design guidelines, safety requirements, and accident analysis. This can prove that K-DEMO and other upcoming fusion energy devices will not result in public evacuations in the future. Philosophically, it is necessary to clarify the soundness of K-DEMO so that stakeholders including the government and the public will accept its construction and operation. As a first step, a conceptual study was performed to stipulate the safety factors, and the regulatory barriers expected under the current Nuclear Safety Act in Korea were examined. The discussion presented in this paper will enable the implementation of fusion systems in consideration of technical guidelines. It will be important to refine and update regulations continually and to define all features with regard to the regulatory framework.

## Discussion

K-DEMO is aimed at demonstrating the engineering feasibility of nuclear fusion with economically attractive net power generation on the scale of hundreds of megawatts. In this paper, we present a preliminary assessment of the safety factors and design requirements to reflect the regulatory environment and to gain social acceptance. The basic specifications and safety factors of KSTAR and ITER were considered for their relevance to K-DEMO and the development of a fusion-compatible regulatory framework. A consensus is needed for safety regulations that support institutional and technological feasibility while promoting social acceptance during the development of fusion plants. New requirements should be devised based on the configurations of specific models and relevant technical baselines that are compatible with fusion systems. To guarantee the soundness of fusion facilities, engineering C&S protocols must be developed on demand. The first step is to prepare institutional measures based on a clear identification of objectives and assessment of associated safety factors. In this paper, we discuss the relationship between safety technology and the regulatory framework from the viewpoint of academics rather than from a position of authority. In future work, we will conduct in-depth research on potential hazards and accident analysis through detailed and specific engineering design and analysis. In addition, we will clarify the philosophical, administrative, and technical gaps between conceptual fusion plants and existing fission plants to realize the development of fusion-compatible guidelines. Such research will help realize a pathway toward a working fusion plant, which is the most challenging technological step in human history to date.

## Supplementary Information

Supplementary Figure S1.
