# Supplementary material for: Preliminary assessment of the safety factors in K-DEMO for fusion compatible regulatory framework
Source: Sci Rep. 2022 May 18;12:8276. doi: 10.1038/s41598-022-12389-w (PMC9117233; doi:10.1038/s41598-022-12389-w)
Supplement: Supplementary file 1 — Supplementary Figure S1. [file 41598_2022_12389_MOESM1_ESM.docx]

***Supplementary Information (Scientific Reports)***

**Preliminary Assessment of the Safety Factors in K-DEMO for Fusion Compatible Regulatory Framework**

# Beom Seok Kim^1,*^, Suk-Ho Hong^2^, Keeman Kim^3^

^1^Department of Mechanical & Automotive Engineering, Seoul National University of Science & Technology, 232 Gongneung-ro, Nowon-gu, 01811 Seoul, Korea

^2^Korea Institute of Fusion Energy, 169-148 Gwahak-ro, Yuseong-gu, 34133 Daejeon, Korea

^3^Korea Institute of Energy Technology, 200 Hyeoksin-ro, Naju, 58330 Jeollanam-do, Korea

^*^Corresponding author

Tel.: +82 2 970 6310

E-mail: kimmbs@seoultech.ac.kr


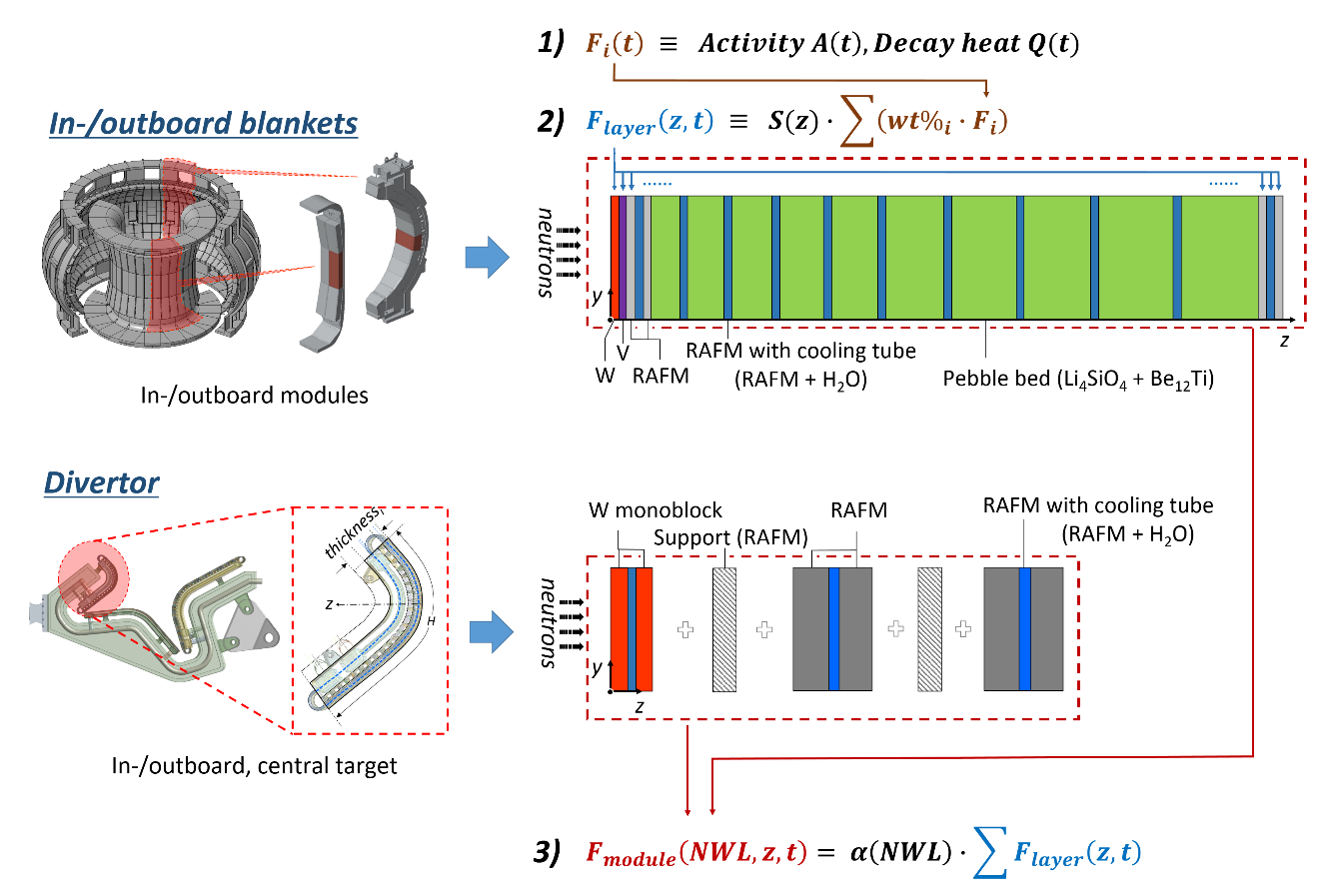


Fig. S1 Process of the radioactivation analysis for a breeding blanket and a divertor component in K-DEMO. *F*(*t*) indicates the radioactivation characteristics (such as radioactivation concentration and decay heat) as a function of time. Subscripts *i*, for the nuclide, the layer and the target module. *S*(*z*) is a derived function for neutron flux attenuation in a solid structure. *α*(*NWL*) is a factor of neutron wall load for corresponding component [1].

**References**

[1] Kim, B. S. et al. Assessment of the activation induced by neutron irradiation in K-DEMO and thermal response under the decay heat. *Fusion Engineering and Design* **146**, 2323 (2019).
